# Financing Long‐Term Services and Supports: Ideas From Singapore

**DOI:** 10.1111/1468-0009.12264

**Published:** 2017-06-06

**Authors:** WAN CHEN KANG GRAHAM, MARCEL BILGER

**Affiliations:** ^1^ Duke‐NUS Medical SchoolSingapore

**Keywords:** long‐term services and support, long‐term care financing, long‐term care insurance, aging

## Abstract

**Policy Points:**

- In Singapore, long‐term services and supports (LTSS) are financed through a mix of public and private sources comprising tiered means‐tested public subsidies and government grants to care providers, charitable donations, voluntary long‐term care insurance, and other private resources.
- Singapore's high take‐up rate of voluntary long‐term care insurance is explained by plans offering partial coverage, medical underwriting, early automatic enrollment, direct debit of insurance premiums, and defined cash benefits.
- Singapore's experience in long‐term care financing is relevant to the US debate, as it provides policy ideas that may be transferable to the American context.
- We recommend maximizing the population's total contribution to voluntary long‐term care insurance by striking the right balance between coverage adequacy and take‐up rate while targeting subsidies to low‐income individuals.

**Context:**

Financing long‐term services and supports (LTSS) for the elderly is a pressing issue in the United States with reforms of long‐term care insurance (LTCI) presently being explored. Singapore, with 65% of residents aged 40 to 83 covered by basic LTCI, including 22% with supplementary LTCI plans, has the highest voluntary LTCI rate in the world. This article contributes to the discourse by presenting the case of LTSS financing in Singapore.

**Methods:**

We first reviewed Singapore's LTSS policies through a comprehensive search of academic papers, governmental reports, parliamentary debate transcripts, print media, and official websites of LTSS providers. We then estimated the LTSS financing mix, conducted an in‐depth analysis of the main policies, and illustrated the financial protection they procure for the elderly using realistic hypothetical scenarios.

**Findings:**

The main principles governing Singapore's LTSS policies are shared responsibility for long‐term care financing, targeted assistance for the poor, centralized governance and administration, separation from other income and housing policies, and limited intergenerational cross‐subsidizing. We estimate the financing mix to consist of out‐of‐pocket spending (40%), government spending (42%), long‐term care insurance (9%), and charitable donations (9%). Assuming a monthly LTSS bill of US$1,545 to US$2,575, between 11% and 19% of LTSS expense is offset by LTCI for severely disabled individuals with basic coverage. Overall, 63% of care recipients are eligible for public subsidies that amount to 20% to 80% of their expenses.

**Conclusions:**

The high take‐up of voluntary LTCI in Singapore is explained by the high prevalence of plans offering partial coverage, medical underwriting, early automatic enrollment, direct debit of insurance premiums, and defined cash benefits. We recommend setting the coverage of voluntary long‐term care insurance plans at levels that maximize the population's total contribution by striking the right balance between coverage adequacy and take‐up rate while targeting subsidies to low‐income individuals.

Financing long‐term services and supports (ltss) for older American adults is a pressing policy issue, whose specific challenges are well known and described in detail elsewhere.^1^, ^2^, ^3^, ^4^, ^5^, ^6^, ^7^ In summary, the demand for LTSS is projected to rise dramatically with the aging of the baby boomers, but the availability of informal care is expected to decrease owing to smaller family size and females’ greater participation in the labor force. The problem is that most Americans are financially ill prepared to pay for expensive formal long‐term care. Two‐thirds of Americans aged 40 and over have not set aside any funds for LTSS.^8^ One of the key policy challenges is to encourage more Americans to obtain financial protection through savings or insurance ahead of time, instead of depending on Medicaid‐funded LTSS after spending down their assets in old age. Elderly Americans, who make up just 10% of Medicaid beneficiaries, already account for roughly 20% of total Medicaid spending (US$438 billion in FY2013).^9^ It is expected that the rising number of old‐old Americans (85 years and older) will drive the growth of Medicaid spending on LTSS at an annual rate of 6% per year between 2012 and 2021.^10^ In contrast, the market for private long‐term care insurance (LTCI) in the United States is languishing, with the number of insurers selling appreciable numbers of LTCI policies having shrunk from 102 in 2002 to 11 today.^11^, ^12^ More than 4 decades after the launch of LTCI in the United States, only 8.1 million Americans have coverage.^13^

Besides France and the United States, Singapore is one of the few countries that employ a combination of tax revenue and voluntary long‐term care insurance for long‐term care financing.^1^, ^5^, ^14^ Singapore stands out because at 65% (among individuals aged 40 to 83),^15^ it has the highest voluntary LTCI rates in the world. In comparison, enrollment rates for people aged 40 years and older are 5% and 15% for the United States and France, respectively.^16^

Even though the United States and Singapore differ in size, culture, and political system, the two countries do share key common characteristics. While Singapore is often called a nanny state, its social policies are founded on the principle of individual responsibility or self‐reliance, one of the United States’ core values. Both countries also favor regulated market solutions and consumer choice. As such, Singapore's experience in long‐term care financing is relevant to the US debate, as it provides policy ideas that may be transferable to the American context.

The issue of how best to help elderly citizens pay for LTSS has preoccupied Singaporean policymakers since the early 1980s, as Singapore is one of the fastest‐aging countries in the world.^17^, ^18^ By 2050, 38% of the population is expected to be over 60 years old, and there will be 2 working‐age adults to every retiree.^19^ Against the backdrop of persistently low rates of national fertility,^20^ the steadily rising level of females’ participation in the labor force,^21^ and increasing longevity—life expectancy at age 65 is 18.9 and 22.1 years for males and females, respectively^22^—Singapore's demand for formal LTSS is expected to rise sharply.

Following the repeal of the Community Living Assistance Services and Supports (CLASS) Act,^23^ the US Senate Commission on Long‐Term Care was convened to identify alternative long‐term care financing solutions. It concluded that there are two potential options—generating more private LTCI options or implementing social insurance—though without coming to a consensus on the best way forward.^3^ In early 2016, the Bipartisan Policy Commission (BPC) put out a report advocating lower‐cost private LTCI products with time‐limited benefits that people can purchase using retirement savings, without penalty. The BPC also recommended encouraging employers to provide LTCI through the workplace on an opt‐out basis.^24^ In efforts to further inform the debate on LTSS financing in the United States, we offer here a comprehensive description of Singapore's long‐term care financing policies, discuss potential adaptations to fit the US context, and propose new ideas that leverage the strengths of both systems.

## Background

### Overview of Long‐Term Services and Supports in Singapore

Singapore is a city‐state located in Southeast Asia. In an area slightly smaller than that of New York City, Singapore has a population of 5.5 million people. The resident population (3.9 million) comprises ethnic Chinese (74.3%), Malays (13.4%), Indians (9.1%), and others (3.2%).^22^, ^25^ Singapore's economy is strong. In 2015, it ranked fourth in gross domestic product (GDP) per capita internationally,^26^ and its government finances are sound, with a long history of budget surpluses.^27^

Singapore spends about 4.2% of its gross domestic product on health care.^28^ Health care financing is characterized by a multipayer system that relies on individual health savings accounts (Medisave), mandatory health insurance (MediShield Life), and general revenues used to reduce the price of health care, especially for low‐income residents. Singaporeans who cannot afford the out‐of‐pocket component of health care can apply for further public assistance (Medifund). This financing system based on the 3Ms (Medisave, MediShield Life, and Medifund) is described in detail elsewhere.^28^, ^29^, ^30^

Eighty percent of inpatient care is delivered by government‐owned hospitals, and 20% of primary care is provided through its polyclinics.^31^, ^32^ With the help of medical social workers, LTSS needs usually are identified at the discharge planning stage of hospitalization episodes. The range of LTSS available in Singapore is comparable to that in the United States. These include home‐based social and medical care, community‐based day activity centers for disabled elderly people with and without dementia, center‐based integrated care for nursing home–eligible elderly still living in their community, chronic sick hospitals, and nursing homes. Hiring foreign domestic workers (FDWs) to care for elderly relatives is a popular alternative.^33^ Women from neighboring countries are employed by Singaporean households as live‐in domestic helpers. In 2011, 12% of all retiree households employed FDWs.^34^ In addition to performing household chores, trained FDWs can assist the elderly in their activities of daily living (ADLs—showering/bathing, dressing, feeding, toileting, transferring from chair to bed or vice versa, and walking or moving on a level surface) and simple maintenance exercises.^35^

The quality of LTSS in Singapore is monitored by the Ministry of Health, which recently drew up official quality standards in consultation with the public.^36^, ^37^ These guidelines focus on ensuring clients’ safety, preserving clients’ dignity, and providing meaningful client‐centered care by a trained staff. Amenities, however, are not regulated. Most nursing homes in Singapore have open wards with 6 or 8 beds in each large, well‐ventilated room. Although instances of poor care or elder abuse by FDWs and private nursing home care staff have been reported, the true prevalence of ill treatment is unknown.

**Figure 1:**
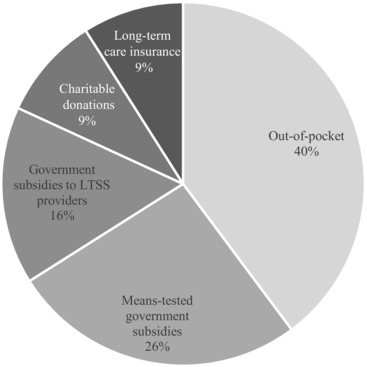
Estimated LTSS Financing Mix in Singapore, by Funding Source Authors’ calculations based on projected and reported figures for 2015. The composition of LTSS spending in 2015 was estimated using the projected LTSS needs of elderly people (aged 65 and over with at least one activity of daily living limitation needing human assistance) generated by a Singapore‐specific long‐term care system dynamics model.^39^ We included estimated expenditures for home‐ and community‐based care, nursing home care, and care by foreign domestic workers. Using estimated proportions of the elderly who are eligible for different levels of means‐tested government subsidies and estimated fees for LTSS, we calculated the total out‐of‐pocket spending and the share of LTSS spending composed of means‐tested government subsidies. With the latter as the base, we estimated the relative sizes of charitable donations and of government subventions to support the operation of LTSS providers. Last, we calculated the share of LTSS spending paid for by long‐term care insurance using an estimated number of elderly claimants.

Official figures for total annual LTSS spending in Singapore are not publicly available. Our calculations (Figure 1) show that LTSS expenditure on people aged 65 and over in 2015 comprised out‐of‐pocket (OOP) spending (40%), government spending (42%), charitable donations (9%), and LTCI (9%). In the United States, it is estimated that the total LTSS expenditure for 2011 came from Medicare (35%), Medicaid (31%), OOP spending (20%), private LTCI (6%), and other sources (6%).^38^

### Shared Responsibility for Long‐Term Care Financing

The policy most emblematic of the individual responsibility principle is that of mandatory savings through the Central Provident Fund (CPF). Like 401(k)/403(b) plans in the United States, these savings belong to the individual account owners without social redistribution. The CPF system follows a life‐cycle model in which Singapore residents (citizens and permanent residents) accumulate savings in their prime to be depleted in retirement. At the end of 2015, 75% of residents aged 25 to 55 years were active CPF members.^40^ Depending on the members’ age, 12.5% to 37% of their total wages is mandatorily transferred into their CPF accounts throughout their working lives. These savings are funneled into 3 accounts: Ordinary, Special, and Medisave, on which their interest is pegged to market interest rates, with guaranteed minimum rates of 2.5% for the Ordinary account and 4% for the Special and Medisave accounts.^41^ Limited preretirement withdrawals are allowed, without penalty, to purchase housing, finance education, and pay medical bills. On average, 60% of total preretirement withdrawals is for housing consumption.^42^ When individuals turn 55, their Ordinary and Special accounts are combined to form Retirement accounts. Dedicated to health care expenses, the Medisave accounts receive 8% to 10.5% of each worker's total wages. Funds in Medisave accounts can be used to pay for inpatient care, for a small selection of ambulatory care services for chronic diseases, and for health‐related insurance within stipulated limits pegged to public subsidy rates.^43^, ^44^

Singapore is a familialist society; that is, its social policies emphasize the primary role of families in providing and financing long‐term care.^45^, ^46^, ^47^, ^48^ The government sends a clear signal that individuals should seek help first from their immediate families by enabling family members to pay one another's hospitalization bills and health insurance premiums using Medisave balances. One's immediate family is defined as one's spouse, children, parents, and grandparents. In order to qualify, grandparents must be residents of Singapore.^49^, ^50^ Under extraordinary circumstances, Medisave can also be used to fund the hospitalization expenses of extended family members such as in‐laws, siblings, uncles, aunts, nieces, and nephews.^51^ Similarly, familial support plays a key role in the United States. Forty percent of American adults are family caregivers,^52^ with 90% of community‐dwelling seniors who need long‐term care receiving informal care from their spouses or children.^53^ The American Association of Retired Persons (AARP) Public Policy Institute estimates that the economic value of informal care provided by families in 2013 (worth US$470 billion) exceeds that of total state and federal Medicaid spending on health care and LTSS combined.^54^

Community organizations form the second pillar of social support in Singapore. In the early 1990s, the term “many helping hands” was coined to describe the prominent role they play in helping the disadvantaged. Under this communitarian approach, the government strongly encourages and supports the charitable activities of voluntary welfare organizations (VWOs), racially organized self‐help groups, and religious groups.^55^, ^56^, ^57^ Indeed, relative to government‐operated organizations, VWOs are believed to be better at providing services and supports because their staff and volunteers are more dedicated to and passionate about their work.^18^

### Targeted Assistance for the Poor

Just as the various Medicaid programs in the United States target aid to those whose incomes are below thresholds based on the federal poverty level, the Singapore welfare system is designed to target subsidies to the truly indigent while discouraging dependency and moral hazard.^58^, ^59^ The welfare system provides assistance for long‐term care financing primarily through subsidies intended to lower out‐of‐pocket costs. Consistent with the residual welfare model, the size of the subsidies is inversely proportional to household income. Monthly per capita household income from work is used to determine both eligibility for aid and the amount of that assistance. In households without income from work, an estimate of the annual income that can be generated by renting out the household residence is used instead. Separately, the Singapore government recognizes that a cohort of residents born on or before December 31, 1949, have not been able to accumulate sufficient CPF funds, owing to low wages and a poorer economy in the country's formative years.^60^ Assistance for the 450,000 members of this “Pioneer Generation” in the form of top‐ups to their Medisave accounts, premium support for the mandatory health insurance MediShield Life, subsidies for outpatient care and medications, and cash assistance for disabled Pioneers with long‐term care needs is provided through earmarked funds.^60^ These are all time‐limited measures, as the cohort will gradually age out of the system.

### Centralized Governance and Administration

Due to Singapore's relatively small size—it does not have a capital and is not divided into states—its governance is highly centralized. Instead, the country is divided into 15 towns, which are managed by local municipalities called town councils.^61^ Unlike states in the United States, town councils are not expected to help fund LTSS for their residents. Instead, all important allocative decisions and policies related to LTSS financing are made by the Ministries of Finance (MOF), Health (MOH), and Social and Family Development (MSF). The MOF allocates government funds earmarked for LTSS, and the statutory boards reporting to MOH and MSF are responsible for determining eligibility and disbursing subsidies.

### Separation From Other Income and Housing Policies

Singapore is a densely populated country with a well‐developed, wheelchair‐accessible public transport system. As many as 80.4% of resident households live in public housing—high‐rise apartments built by the Housing Development Board (HDB).The remainder live in private apartments or landed property.^62^ HDB apartments built since 1985 incorporate barrier‐free designs. Older apartment buildings have been retrofitted with features such as grab bars and antislip tiles in the bathrooms, ramps, and lifts that stop at every floor. HDB's future plans are to build “smart” homes equipped with remote sensing and alert systems for the elderly.^63^

Income and housing policies in Singapore are closely intertwined, but not explicitly coordinated, with policies concerning long‐term care financing. Since large portions of CPF savings can be used to purchase a home, 90.8% of Singapore resident households own their residential property.^64^ Then as property prices rise over time, one's share of residential property as part of one's total household assets rises correspondingly. At the end of 2000, residential property represented 51% of total assets for Singaporean households, compared with 28% in the United States.^65^ Thus, many lower‐income households in retirement are “asset rich and cash poor.”^66^, ^67^

To rectify this situation, the government introduced measures to cap the amount of CPF savings that can be used for property financing and to increase the amount of CPF savings—the retirement sum—that must be set aside at age 55.^68^, ^69^ A lease buyback scheme (similar to reverse mortgages) has been implemented to allow retirees to tap their housing wealth while continuing to live in their properties.^70^, ^71^ The Housing and Development Board also began building elder‐friendly studio apartments with 30‐year leases and offering cash incentives to encourage older people to “right‐size” into smaller apartments, thereby unlocking home equity in the process. While these measures help increase retirement income, a portion of which may be used to pay for LTSS, none of them addresses the issue of long‐term care financing directly.

### Limited Government‐Administered Intergenerational Cross‐Subsidizing

Under the current political climate, new social insurance programs are unlikely to be created in the United States. Similarly, the Singapore government has also resisted adopting a social insurance–based financing system. Specifically, it rejects the form of direct intergenerational cross‐subsidization inherent in pay‐as‐you‐go (PAYG) pension systems. It is not opposed, however, to using general revenues to subsidize LTSS use by low‐income residents. Thus, some degree of government‐administered intergenerational transfer exists. Even so, public subsidy is one of several sources of LTSS financing in Singapore. The Singapore system also relies on LTSS financing through private income and savings, private long‐term care insurance (ElderShield), charitable donations, and informal intergenerational transfers within families. To limit the amount of intergenerational transfer it administers, the government seeks to finance public subsidies using income generated from endowment and trust funds whenever possible. It achieves this by setting aside budget surpluses to create or top up such funds regularly. Although capital endowments come from the current generation, such arrangements are not strictly PAYG, as these funds will also benefit, in the future, those individuals whose taxes helped to seed these endowments today.^72^

The purpose of these schemes (Table 1) is to ensure that government financing for LTSS is sustainable. Forecasting future long‐term care expenditure is, however, extremely challenging, given the multitude of variables—disability rate, duration of disability, availability of informal care, and cost of LTSS—for which good estimates are lacking. Whether the government and individuals have set aside sufficient funds remains to be seen.

**Table 1:** Financing Schemes for Retirement, Health and Long‐Term Care in Singapore[Link], [Link]

Focus	Program	Description
Retirement	CPF Ordinary Account	Mandatory savings for housing, insurance, investment, and education Proportion of wages saved decreases progressively with age, from 23% (≤ 35‐year‐olds) to 1% (> 65‐year‐olds)
	CPF Special Account	Mandatory savings for old age and investment in retirement‐related financial products Proportion of wages saved decreases progressively with age, from 6% (≤ 35‐year‐olds) to 1% (> 65‐year‐olds)
Health care (3Ms)	Medisave	Mandatory savings to pay for hospitalizations and approved health‐related insurance Proportion of wages saved increases progressively with age, from 8% (≤ 35‐year‐olds) to 10.5% (> 65‐year‐olds)
	MediShield Life	
	Basic	Mandatory health insurance administered by CPF board
	Supplemental	Voluntary private health insurance to augment MediShield Life
	Medifund	A government safety‐net program to help indigent patients with their medical bills
Long‐term care	ElderShield	
	Basic	Voluntary long‐term care insurance with automatic enrollment feature Default coverage: $400/month × 72 months
	Supplemental	Voluntary additional long‐term care insurance to augment Basic coverage Optional coverage $500 to $5,000/ month × from 10 years to lifetime
	Medifund Silver	A government safety‐net program to help indigent elderly patients with their medical and long‐term care bills
	IDAPE	A government financial assistance program for indigent elderly people who were not eligible for ElderShield
	PioneerDAS	A government financial assistance program for disabled elderly Singaporeans born on or before December 31, 1949 (the Pioneer Generation)
	FDW Grant	A government grant for low‐ and middle‐income families that need to hire FDWs to help care for disabled family members, $120/month
	FDW Levy Concession	All families that need to hire FDWs to help care for disabled family members pay the concessionary FDW levy of $60/year instead of the full FDW levy of $265/year

Abbreviations: CPF, Central Provident Fund; IDAPE, Interim Disability Assistance Program for the Elderly; PioneerDAS, Pioneer Generation Disability Assistance Scheme; FDW, Foreign Domestic Worker.

^a^Information obtained from the websites of the Central Provident Fund Board (www.cpf.gov.sg) and the Ministry of Health (www.moh.gov.sg).

^b^Dollar amounts are given in Singapore dollars. The exchange rate (1 SGD = 0.7356 USD) is as published on the *New York Times* website on September 13, 2016.

## Long‐Term Care Financing Policies in Singapore

### Long‐Term Care Insurance

The United States has had a long‐term care insurance market since 1974.^73^ A prototype LTCI policy that served as a template for policies that are common today was described by Meiners in 1983.^74^ In comparison, LTCI is a relatively new financial instrument in Singapore.

ElderShield was developed in 2001 and implemented in 2002 after the government concluded that financial protection against catastrophic spending on severe disability, which is a low‐probability, high‐cost event, is best provided through insurance.^75^ Currently, among Singapore residents aged 40 to 83, 65% are covered by basic ElderShield plans, and 22% have supplemental coverage.^15^ The basic level of ElderShield coverage pays a fixed cash benefit of S$400 (US$294) per month for 6 years, regardless of the actual level of disability beyond the minimum level needed to qualify for a claim. Supplemental ElderShield plans are optional add‐ons that can be bought only by those already covered by the basic ElderShield plan. These plans pay out higher monthly cash benefits, ranging from S$500 (US$368) to S$5,000 (US$3,678) for longer periods, ranging from 6 years to life.^76^, ^77^, ^78^ A 90‐day waiting period between claim submission and benefit payout applies to both types of LTCI plans.

Healthy Singapore citizens and permanent residents between the ages of 40 and 65 without serious preexisting disability or serious chronic medical conditions are eligible for basic ElderShield coverage. All residents with Medisave accounts are automatically enrolled into this default plan when they turn 40. Health declaration forms that accompany the enrollment notification packets help screen out individuals with severe disabilities.^79^ Exact figures for the prevalence of disability at different ages are not available, but the official estimate places the disability prevalence rate of the resident population at 3%.^80^

New enrollees are randomly assigned to one of the 3 private, for‐profit insurers—NTUC Income, Great Eastern Life, or AVIVA—contracted to the Singapore government to offer both basic and supplemental ElderShield plans. This method of choosing vendors through competitive bidding is similar to that used to implement the Federal Long‐term Care Insurance Program (FLTCIP).^81^ These insurers in Singapore are large, reputable companies that offer a wide range of insurance products in addition to ElderShield.^82^, ^83^, ^84^ Their contracts are subject to renewal approximately every 5 years, at which time these insurers are required to return excess premiums collected (relative to payout) to policyholders in the form of premium rebates. While the set of terms and conditions for basic ElderShield coverage is the same for all, these insurers have considerable latitude in the design and marketing of their supplemental plans, subject to regulation from the government.^85^ As a result, enrollees can choose among different premium payment durations (fixed or lifetime), different additional cash benefits (for dependents, for rehabilitation, and in the event of death), different claim criteria (2 instead of 3 ADL limitations), and different payout periods (10 years, 12 years, or lifetime).^86^

New enrollees may opt out within 3 months of enrollment. When ElderShield was launched in 2002, Medisave account holders aged 40 to 69 were automatically enrolled, and 33% of those enrolled in this manner dropped out in the first year.^87^ By 2006, the opt‐out rate of new enrollees had dropped to 14%.^88^ Those who opt out may reenroll later, subject to underwriting. Enrollees may also switch insurers at any time. There are no penalties for new enrollees if they switch within the initial 90‐day opt‐out period. Those who switch later will be subject to underwriting by the new insurer, and the premiums paid to the original insurer will be forfeited.^89^ Finally, policyholders may obtain basic and supplemental coverage from different insurers. That is, they are free to purchase supplemental plans from any of the 3 insurers at any time before they turn 65, irrespective of their basic ElderShield insurer assignment. Insurers may contact new policyholders by phone directly or through third‐party marketers to sell their supplemental plans. These plans conduct their initial risk screening through a short questionnaire. This screen flags those applicants whose risk profiles warrant medical examination. Rejection rates for supplemental plans are not published. As long as they do not already have serious ADL limitations, automatically enrolled individuals will retain basic ElderShield coverage even if they have preexisting medical conditions.

In principle, all healthy 40‐year‐old residents without serious ADL limitations have the same premiums when they are automatically enrolled in the default basic ElderShield plans. These premiums, which were last adjusted in 2007, amount to S$175 (US$129) and S$218 (US$160) per year for males and females, respectively. Annual premiums are payable until age 65. Residents who are not part of automatic enrollment (ie, those without Medisave accounts) may apply for coverage on their own. Age‐ and risk‐rated annual premiums for ElderShield are determined at the time of enrollment. Premiums for supplemental plans range widely. For healthy 40‐year‐old males in 2016, annual premiums ranged from S$217 (US$160) to S$3,134 (US$2,305). These premium levels correspond to lifetime cash benefits of S$500 (US$368) to S$5,000 (US$3,678) per month.^76^, ^77^ Medical underwriting applies to supplemental plans. Those who enroll at older ages or have preexisting medical conditions such as diabetes face higher premiums. Medisave funds may be used to pay ElderShield premiums, with an annual withdrawal limit of S$600 (US$441) per enrollee.^85^ Enrollees with insufficient Medisave funds may tap the Medisave accounts of immediate family members or pay out of pocket. Annual premiums in excess of S$600 (US$441) must be paid out of pocket.

Since ElderShield is not tied to employment, coverage is maintained regardless of the policyholders’ employment status, although coverage can be lost if the premiums are not paid. Policyholders are granted a 75‐day grace period from the premium due date, after which the policies lapse. However, policyholders may apply for reinstatement of coverage within 180 days after the end of the grace period. A nonforfeiture clause in more comprehensive supplemental plans allows policyholders who have paid a stipulated minimum number of premiums to claim prorated benefits instead of losing coverage altogether if they are unable to continue paying premiums.^86^ Data on the prevalence of lapsed ElderShield plans are not available.

Similar to the United States, Singapore's regulations allow for the amendment of premium and benefit levels of active policies, although the phenomenon of rapidly rising premium rates has not been observed. In fact, premium rebates have been issued twice in the past 14 years.^90^ ElderShield plans, both basic and supplemental, do not have cash‐surrender value. Unlike the United States, none of the LTCI policies offered in Singapore provides inflation protection.

Because people with serious preexisting ADL limitations and those aged 70 and older in 2002 were ineligible for ElderShield at its launch, the government created a parallel cash subsidy program, the Interim Disability Assistance Programme for the Elderly (IDAPE), to ensure that they also would have access to benefits. IDAPE benefits are subject to the same disability requirements as ElderShield and are means tested. Eligible individuals receive between S$100 and S$250 (US$74 and US$184) per month for a maximum of 6 years.^91^

ElderShield enrollees may claim benefits at any age after enrollment. Benefits for basic ElderShield plans are payable when people are unable to perform 3 out of 6 basic activities of daily living (ADLs), as determined by MOH‐accredited physicians.^92^, ^93^ Simulations performed using a Singapore‐specific long‐term care system dynamics model^39^ projected that in 2015, 3% of people over 65 had 3 or more ADL limitations requiring human assistance. Assessment for eligibility also takes into account the claimant's cognitive status. Individuals with cognitive deficits (eg, dementia) are graded as dependent even when they are physically capable of ADLs.^94^ There are no restrictions on how the cash benefits are used. ElderShield claimants are not required to comply with any stipulated care plans or seek care from “approved” providers.

One important difference between the two countries’ LTCI policies is the types of benefits. Most American LTCI policies offer service benefits, while ElderShield plans offer only defined cash benefits.^95^, ^96^ In the United States, only a few insurers offer the option of claiming LTCI benefits in cash. When they do, the amount of cash that can be disbursed is based on the value of the original service benefit that can be claimed. Usually, only a fraction of the service benefit allowance can be received in cash.^96^

Another major difference is one of expectations. In the United States, LTCI is expected to cover most, if not all, of LTSS expenses. In contrast, basic ElderShield coverage is meant only to help defray the cost of paying for formal long‐term care. Singaporeans who can afford it have the option of purchasing higher levels of coverage with benefits large enough to cover most of their expected LTSS expenses, but a high level of coverage is not the default. This reflects not only the government's aims of making the default option affordable and promoting a high uptake of LTCI but also a concern of not nudging individuals into a plan that might be deemed excessive by those who are able to rely on informal care or other financial resources.

The ElderShield LTCI scheme as described is currently undergoing a major review following the rollout of mandatory MediShield Life, an enhanced version of the preexisting and nonmandatory catastrophic health insurance MediShield.

### Subsidies Through Public Funds

While public assistance for LTSS in the United States is administered mostly through Medicaid programs, public funding streams for long‐term care financing in Singapore flow in 3 ways: grants to lower operating costs of VWOs, reimbursements paid to service providers for the care of subsidized patients (hereafter referred to as means‐tested subsidies), and other subsidies disbursed directly to LTSS recipients.

Grants to VWOs can fund up to 50% of their operating costs and up to 100% of their capital costs.^97^ In order to quickly ramp up the provision of nursing home care, the government rolled out the Build‐Own‐Lease program in 2012.^98^ Under this scheme, the government bears the full capital cost of setting up nursing homes. These facilities are built and owned by the government but are leased out for operation by VWOs or private companies.^99^ By lowering the LTSS providers’ costs, the government is indirectly lowering the out‐of‐pocket costs to care recipients.

Means‐tested subsidies are another avenue of public funding for long‐term care. Even when services are provided by VWOs, recipients are expected to pay service fees. Subsidized LTSS recipients pay a fraction of the full‐fee price of care, while the government reimburses LTSS providers the remainder. In other words, the out‐of‐pocket expenditure for someone receiving LTSS from a VWO is equal to the portion of the bill that remains after the government grants and means‐tested government subsidy have been applied. Because the subsidies are calculated based on a universal set of standard prices (also called “norm costs”) for LTSS, subsidy sizes are uniform for recipients within each subsidy level. In order to meet the demand for LTSS, the government is making such subsidies increasingly “portable” so that private LTSS providers can be reimbursed in the same manner for a subset of their clients.^98^

Means testing is carried out at the household level. Specifically, it is based on the monthly per capita household income (MPCHI), or the income from work of all the care recipient's immediate and distant relatives living at the same address. In principle, all those living in households with an MPCHI of S$2,600 (US$1,913) or below are eligible for some level of public subsidy. To put things in context, households with an MPCHI of S$650 (US$478) or lower are eligible for urgent public assistance.^100^ Using this as the de facto poverty line—an official one does not exist—public subsidies are available for households whose MPCHI is up to 4 times the poverty line. Given that the median MPCHI is S$2,500 (US$1,839),^101^ roughly half of Singapore's resident households are eligible for subsidized care. Families applying for additional public assistance are subject to family‐level means testing, in which case the incomes of non‐co‐resident family members are also included.^102^ Household‐level means testing is in line with Singapore's governing principle of relying on families first, as retirees living with economically successful relatives tend to receive smaller public subsidies than do retirees who live on their own or with less well‐off relatives. Since roughly two‐thirds of elderly Singaporeans co‐reside with their children,^103^ a substantial number of retirees are affected by such means testing. Adult children who live apart from their elderly parents are not completely free of responsibilities. Singapore's filial responsibility law, the Maintenance of Parents Act,^104^ allows parents to sue adult children for maintenance, even though few do.^105^

Public subsidy levels are inversely proportional to the MPCHI. Singapore citizens receive higher rates of subsidies than permanent residents do. The levels of subsidy for citizens range from 30% to 80% for HCBS and from 20% to 75% for nursing homes. (Corresponding subsidy levels for permanent residents are 15% to 55% and 10% to 50%, respectively.^106^) Thus, recipients with an MPCHI of S$1,801 (US$1,325) to S$2,600 (US$1,913) bear 70% to 85% of the full‐fee prices of care, whereas those with an MPCHI below S$701 (US$516) bear 20% to 45%. Based on data from the General Household Survey of 2015^63^ and the Families and Households Report for 2010‐2014,^107^ we estimated that 63% of community‐dwelling Singaporeans aged 65 years and over are eligible for subsidized LTSS and that 26% of them qualify for the highest level of subsidy. Medifund Silver, a safety‐net program of last resort, is available for those who cannot afford the postsubsidy expenses.^108^ In addition, the Seniors’ Mobility and Enabling Fund (SMF) provides subsidies for assistive devices, home health care consumables such as feeding tubes and diapers, and transportation to day service centers.^109^

The third way that public funds are used to finance LTSS is by making grants directly to care recipients. Specifically, the government hands out cash (S$120 per month, US$88) to households that choose to hire FDWs. This FDW grant offsets approximately one‐tenth of the monthly FDW expense. It is means tested, and the care recipient has to have at least 3 ADL limitations as determined by standardized medical assessment.^110^ In addition, the government also uses tax expenditure to lower the cost of hiring FDWs. The tax for employing an FDW (termed the “monthly FDW levy”) is less than a third of the usual rate (S$60 instead of S$265 per month, US$44 instead of US$195).^111^ Most recently, the government launched the Pioneer Generation Disability Assistance Scheme (PioneerDAS), which provides an additional S$100 (US$74) per month to senior citizens with 3 or more ADL limitations to help defray LTSS costs.^112^

### Charitable Donations

In the United States, the majority of long‐term care providers are for‐profit.^113^ The opposite is true in Singapore, where most social welfare services, including LTSS, are provided by VWOs.^114^ At last count, there were a total of 2,217 registered VWOs.^115^ Ninety‐one of those registered under the Social and Welfare sector are identified as providers of elder care services (homemaking, home personal care, meal delivery, center‐based day care), and 30 of those registered under the Health sector are listed as providers of institutional care (nursing homes and chronic sick hospitals), home medical services, and day rehabilitation.^116^ Approximately two‐thirds of the 12,000 nursing‐home beds in Singapore are managed by VWOs.^117^ Many of these VWOs are religion based, but others were started by secular communities or through private philanthropy.^115^

Some VWOs are large and operate throughout the island, whereas others are small and serve specific communities near their facilities. There is a competitive market for home and community‐based services (HCBS), as recipients may select from a number of providers located near their homes and switch providers at will. Nursing home beds, however, are in short supply. Most beds in VWO‐operated nursing homes are subsidized and are almost fully occupied. Recipients who are not eligible for public subsidies or who are unable to wait for bed openings tend to use private nursing homes. Although nursing homes and other LTSS providers are regularly audited by the MOH, detailed objective quality ratings such as those presented on Medicare's Nursing Home Compare website^118^ are not publicly available. The government helps maintain service quality by equipping VWOs with “best practice” operational guidelines^119^ and encouraging their adherence to those models through licensing requirements, training, and performance‐based funding.^114^, ^120^

VWOs are expected to raise funds to augment their income from government grants and service fees. Those VWOs that are better at attracting donations have larger operating budgets and can, at their own discretion, use their income to further lower the price of LTSS for some or all of the senior citizens under their care. We estimate that 9% of total LTSS spending in 2015 came from charitable donations (see Figure 1).

## Singapore's LTSS Financing Policies at Work

We now illustrate the mechanisms by which these financing policies help offset LTSS out‐of‐pocket expenditure for 6 simplified hypothetical recipients (Table 2). These cases were generated using 2 levels of disability (mild: 1 to 2 ADL limitations; and severe: 3 or more ADL limitations) and 3 levels of informal care availability (full time: a healthy retired spouse; limited: a working child; and none: recipient lives alone). For example, a person suffering from weakness on one entire side of the body (hemiparesis) who needs only supervision or minimal assistance with activities of daily living is considered mildly disabled and ineligible for ElderShield, IDAPE, and PioneerDAS benefits. But a person who is incontinent, is wheelchair dependent, and requires maximum assistance for bathing and dressing is considered severely disabled and is therefore eligible for insurance payouts and other benefits.

**Table 2:** Estimated Monthly LTSS Expense (Full, Unsubsidized Fee) by Level of Disability and Availability of Informal Care^a^

			Quantity of LTSS Recommended per Month								
			(Unit Fee)								
Hypothetical Long‐Term Care Recipients,			Home Medical	Home Nursing	Home Occupational Therapy^b^	Home Physical Therapy^b^	Home Personal Care^c^	Meal Delivery	Transport	Senior Day Care Regular^d^/ Intensive^e^ (Regular Full Day: S$55/day;	Estimated Average Monthly LTSS Expense^f^ in SGD
Level of Disability and Level of Informal Care			(S$183/session)	(S$79/session)	(S$113/session)	(S$113/session)	(S$26/hour)	(S$5/meal)	(S$45/2‐way trip)	Intensive Full Day: S$70/day)	(1 SGD = 0.7356 USD^g^)
I	1‐2 ADL limitations, helped by healthy retired spouse		n/a	n/a	4	4	16	12	n/a	n/a	$1,378^h^
II	1‐2 ADL limitations, helped by full‐time working child		n/a	n/a	n/a	n/a	n/a	n/a	20	Regular: 20 days/ month	$2,000
III	1‐2 ADL limitations, no informal care available		n/a	1	n/a	n/a	n/a	36	20	Regular: 20 days/ month	$2,266
IV	3+ ADL limitations,^i^ helped by healthy retired spouse		1	2	4	4	36	56	n/a	n/a	$2,468^h^
V	3+ ADL limitations,^i^ helped by full‐time working child		1	2	n/a	n/a	n/a	n/a	20	Intensive: 20 days/ month	$2,641
VI	3+ ADL limitations,^i^ no informal care available										
	VWO‐operated^j^: low ‐ high		n/a	n/a	n/a	n/a	n/a	n/a	n/a	n/a	$2,100 ‐ $3,500^k^
	Private^l^: low ‐ high		n/a	n/a	n/a	n/a	n/a	n/a	n/a	n/a	$3,500 ‐ $8,300^k^

^a^The estimates were generated from multiple sources. For home care, we relied on an anonymous survey of home care providers. Representatives of both VWOs and private providers were surveyed. The survey was approved by the IRB of the National University of Singapore. We mailed the questionnaire to 8 providers that the Agency of Integrated Care identified as able to offer a comprehensive range of home care services. The questionnaire contained vignettes describing two 75‐year‐old female care recipients with chronic right‐sided weakness, one with 2 ADL limitations, and the other with 5 ADL limitations. For each disability level, 3 possible informal care availability levels were described: high (helped by 77‐year‐old healthy, retired husband), moderate (helped by 50‐year‐old unmarried daughter who works full time), and low (recipient lives alone). We assumed that severely disabled recipients who live alone would require nursing home care and omitted that vignette from the survey. We asked respondents to indicate the types and quantity of home care services that they would recommend in each of the 5 remaining vignettes. Respondents were specifically asked to assume that an FDW would not be hired and to make their best recommendations without regard to the recipients’ financial status. Respondents were also asked to provide the unsubsidized full fees their organizations would charge for the recommended services. Based on the responses, we were able to work out the recommended types, quantities, and average unsubsidized fees of home care services for each vignette. Fees for day care and nursing home care were obtained through direct inquiry made to providers of the respective services.

^b^Typically, only the first 18 home‐based therapy sessions are subsidized. Care recipients may appeal for more subsidized home‐based sessions, pay full‐fee for home therapy, or transfer to subsidized center‐based therapy.

^c^Home Personal Care services include bathing, maintenance exercises, light housekeeping, meal preparation, grocery shopping, medical escort, and companionship.

^d^Regular senior day care refers to community‐based supervision and support. Sociorecreational activities, maintenance therapy, and meals are provided.

^e^Intensive senior day care refers to community‐based care for bed‐ or wheelchair‐bound nursing home–eligible individuals with high care needs.

^f^To obtain the monthly totals, units of weekly services are multiplied by 4 and the corresponding unit fees. Similarly, units of daily services are multiplied by 20 and the corresponding unit fees.

^g^All average and total fees are expressed in Singapore dollars. The exchange rate stated is as published on the *New York Times* website on September 13, 2016.

^h^Expenditures are estimated based on the assumption that senior day care is not used when informal care availability is high.

^i^People with severe cognitive impairment are eligible for ElderShield benefits and other subsidies directed at people with 3+ ADL limitations whether or not they are actually physically impaired.

^j^VWO‐operated nursing homes serve primarily subsidy‐eligible recipients. Shared accommodations consist of 2‐,3‐,4‐,6‐,8‐,12‐, or 24‐bed non‐air‐conditioned open wards.

^k^Estimated expenditures include room and board, and recurring charges for consumables (eg, diapers and milk feeds), ambulance transport to medical appointments, on‐site medical care, and goods and services tax.

^l^Estimated expenditures are for private nursing homes that are not part of the portable subsidy scheme. Shared accommodations consist of 1‐,2‐,3‐,4‐,5‐, or 8‐bed open wards. Rooms are generally air‐conditioned except for the 8‐bed open wards.

To estimate the magnitude of LTSS expenses for the recipients in these hypothetical scenarios, we gathered the expert opinions of medical social workers, rehabilitation therapists, and care managers currently working for LTSS providers. Based on written descriptions of the hypothetical scenarios and without making any assumptions about the recipients’ income, the experts were asked to list the types and quantities of LTSS that they would recommend. They were also asked to provide, whenever possible, the full fee unit price (unsubsidized price) of each recommended service. Because the sets of available services and the corresponding eligibility criteria varied among HCBS providers, this exercise yielded a range of LTSS arrangements for each scenario. We integrated the different recommendations and created 6 plausible hypothetical care packages. The average monthly LTSS expense for each package was estimated using both expert‐supplied and publicly available price lists.^121^ The types and quantities of LTSS recommended and the estimated monthly full‐fee expense are shown in Table 2. In the case of nursing home care expense, the estimated monthly fee includes room and board, as it does in the United States. LTSS fees are slightly lower in Singapore, where, for example, the unsubsidized daily rate for senior day care ranges from S$55 to S$80 (US$40 to US$60), compared with the median rate of US$68^122^ in the United States. As expected, LTSS expense is the lowest for people with 1 to 2 ADL limitations who have ready access to informal care. The expense then rises with increased disability and decreased availability of informal care.

Based on these figures, we estimated the out‐of‐pocket LTSS expense for 70‐year‐old females with 3 or more ADL limitations who are currently receiving services. In this analysis, we estimated the outcomes for 3 types of ElderShield enrollees: those with basic, Medisave Supplemented, or Medisave and OOP Supplemented coverage. Those who do nothing after being automatically enrolled will have basic coverage by default. Those individuals who choose to direct the maximum allowable Medisave withdrawal amount (S$600 or US$441) to ElderShield have Medisave Supplemented coverage. We assumed that they always allocate S$600 (US$441) of Medisave funds to buy as much coverage as is possible for their age at enrollment. A third group of people, comprising those who increase their premium level beyond the Medisave withdrawal limit by topping it up with a proportion of their disposable income at time of enrollment, have Medisave and OOP Supplemented coverage. We assumed that the value of such cash top‐ups is equivalent to 1% of the annual average per capita household income in 2007, which amounts to S$256 (US$188).^123^ In this simulation, we also assumed that everyone enrolled in 2007, when supplementary plans first became available.

Based on the assumptions laid out in Table 3, we then calculated the cash benefits that can be claimed by each type of ElderShield enrollee. Table 4 demonstrates the offsetting effects of subsidies and different levels of ElderShield coverage on monthly OOP expenses as expressed in Singapore dollars and as percentages of the full fees. As expected, higher levels of ElderShield coverage correspond to lower OOP expenses for recipients who are ineligible for subsidies. Subsidies alone reduce OOP expense by up to 80% for eligible recipients without ElderShield coverage. In combination, the reduction in OOP expense can be substantial. To illustrate, consider the recipients in scenario IV (see Table 4). The OOP expense of people with basic coverage receiving a 30% subsidy is nearly halved after both subsidies and ElderShield benefits are applied. For the poorest people with basic coverage, the combination of an 80% subsidy and ElderShield benefits brings OOP expenses down to just 3% of the full fee.

**Table 3:** Assumptions About Premiums and Benefits Used in Estimating the Out‐of‐Pocket Expenses of 70‐year‐old Females[Link], [Link], [Link]

	ElderShield Basic	Medisave Supplemented	Medisave and OOP^d^ Supplemented
Premium/year	$1,156.39^e^	$600^f^	$856^g^
Cumulated premium^h^	$5,796.95	$12,000	$17,120
Monthly cash benefit	$400	$523	$653
Payout period	72 months	Lifetime payout	Lifetime payout

^a^We assumed that all these females enrolled in ElderShield (basic or supplemented) plans at age 61 in 2007.

^b^All premiums and benefits are expressed in Singapore dollars. The exchange rate (1 SGD = 0.7356 USD) is as published on the *New York Times* website on September 13, 2016.

^c^Calculations are based on NTUC Income premium tables; benefit levels are proportional to premiums paid and are prorated when the exact premium amounts do not appear on the premium table. All plans are assumed to be fully prefunded.

^d^OOP stands for the out‐of‐pocket portion of premiums of supplemented ElderShield plans.

^e^We modeled 61‐year‐old females who paid $1,156.39/year under an accelerated 5‐year schedule.

^f^We modeled enrollees who met the maximum Medisave withdrawal limit for ElderShield premiums. For this group, the annual premium is $600. According to the premium table, these enrollees should make 20 annual premium payments.

^g^We modeled enrollees who, in addition to meeting the Medisave withdrawal limit, further topped up their premiums with OOP. OOP is assumed to be equal to 1% of the annual average per capita household income in 2007 (OOP = $256). Thus for this group, the annual premium is $856. According to the premium table, these enrollees should make 20 annual premium payments.

^h^This is the total amount of insurance premium to be paid throughout the pay‐in period.

**Table 4:** Estimated Average Monthly Out‐of‐Pocket Expenses for 70‐year‐old Females With 3+ ADL Limitations, After Applying Subsidies and ElderShield Benefits, in 2016^a^

			Estimated Out‐of‐Pocket Expenses for Home‐ and Community‐Based Care by Government Subsidy Levels, Based on Per Capita Household Income (% of Fees Subsidized)^c^					
			≥ $2,601	$1,801−$2,600	$1,601−$1,800	$1,101−$1,600	$701−$1,100	$0−$700
Level of Informal Care Available		Size of ElderShield Benefits^b^	(0%)	(30%)	(50%)	(60%)	(75%)	(80%)
IV)	Help from healthy retired spouse							
	Uninsured	$0	$2,468	$1,728	$1,234	$987	$617	$494
			100%	70%	50%	40%	25%	20%
	ElderShield Basic	$400	$2,068	$1,328	$834	$587	$217	$94
			84%	54%	34%	24%	9%	4%
	Medisave Supplemented	$523	$1,945	$1,205	$711	$464	$94	$0
			79%	49%	29%	19%	4%	0%
	Medisave and OOP Supplemented	$653	$1,815	$1,075	$581	$334	$0	$0
			74%	44%	24%	14%	0%	0%
V)	Help from full‐time working child							
	Uninsured	$0	$2,641	$1,849	$1,321	$1,056	$660	$528
			100%	70%	50%	40%	25%	20%
	ElderShield Basic	$400	$2,241	$1,449	$921	$656	$260	$128
			85%	55%	35%	25%	10%	5%
	Medisave Supplemented	$523	$2,118	$1,326	$798	$533	$137	$5
			80%	50%	30%	20%	5%	0.2%
	Medisave and OOP Supplemented	$653	$1,988	$1,196	$668	$403	$7	$0
			75%	45%	25%	15%	0.3%	0%
VIa)	No informal care, VWO nursing homes^d^							
	Uninsured	$0	$2,100	$1,680	$1,260	$1,050	$840	$525
			100%	80%	60%	50%	40%	25%
	ElderShield Basic	$400	$1,700	$1,280	$860	$650	$440	$125
			81%	61%	41%	31%	21%	6%
	Medisave Supplemented	$523	$1,577	$1,157	$737	$527	$317	$2
			75%	55%	35%	25%	15%	0%
	Medisave and OOP Supplemented	$653	$1,447	$1,027	$607	$397	$187	$0
			69%	49%	29%	19%	9%	0%
VIb)	No informal care, private nursing homes^d^							
	Uninsured	$0	$3,500	n/a	n/a	n/a	n/a	n/a
			100%					
	ElderShield Basic	$400	$3,100	n/a	n/a	n/a	n/a	n/a
			89%					
	Medisave Supplemented	$523	$2,977	n/a	n/a	n/a	n/a	n/a
			85%					
	Medisave and OOP Supplemented	$653	$2,847	n/a	n/a	n/a	n/a	n/a
			81%					

^a^All dollar values are expressed in Singapore dollars. The exchange rate (1 SGD = 0.7356 USD) is as published on the *New York Times* website on September 13, 2016.

^b^Fixed defined cash benefits for 70‐year‐old females are shown in this column.

^c^Government subsidy levels are shown in the row below. The government defines "norm costs" for each type of LTSS presented in Table 2. The subsidies are applied to these "norm costs" and not to any additional markups. We assumed that the full, unsubsidized LTSS fees calculated in Table 2 consist only of "norm cost" values. Dollar values in the rest of the table represents the out‐of‐pocket payments that care recipients face after ElderShield benefits and government subsidies have been applied. The percentage value below each dollar value shows the out‐of‐pocket payment expressed as a percentage of the full, unsubsidized fee. We assumed that the recipients are Singapore citizens and applied the corresponding subsidy levels.

^d^The lower estimates of nursing home expenditure depicted in Table 2 were used in these calculations.

Our calculations confirm that Singapore's LTSS financing system is designed primarily with the bottom half of the income distribution—and especially the poor—in mind. The means‐tested, tiered subsidy system allows the majority of elderly Singaporeans to be eligible for subsidies, and the low‐cost basic ElderShield plan provides further financial protection for eligible claimants. Elderly Singaporeans in the upper half of the income distribution, however, bear the burden of LTSS expenditure almost entirely on their own, through a combination of ElderShield benefits, personal assets, and family support.

Even though the government's official policy stance is one of self‐reliance and familial responsibility, public spending already constitutes a substantial portion (42%) of total LTSS expenditure (see Figure 1). With family size shrinking due to a persistently low fertility rate, the number of people who can realistically rely on family members for informal care and financial support will decline. Financing strategies facilitated by the pooling and sharing of Medisave funds within families will become less effective. As a result, the government is under pressure to adopt alternative policies to cope with the upcoming rise in demand for public assistance. The government's principle of individual and familial responsibility has already shifted to that of shared responsibility within society at large, as evidenced by the new MediShield Life insurance scheme, which is a mandatory version of the previous MediShield insurance scheme.

While the LTCI take‐up rate is high in Singapore, the level of cash benefit is low for most enrollees. When the ElderShield scheme was first introduced in 2002, policymakers deemed that the premiums associated with the monthly benefit of S$400 (US$294) would not be overly burdensome for most people. Even though supplementary plans were introduced in 2007, two‐thirds of current ElderShield policyholders remain insured at the default basic level. The current default monthly benefit of S$400 is low relative to the LTSS expenses we estimated in Table 2 and even relative to the cost of hiring an FDW, which exceeds S$1,000 (US$736) per month. However, it is important to keep in mind that the system was not designed to fully cover LTSS expenses in efforts to limit dependency and moral hazard but instead to promote shared financial responsibility. Another concern is that the 6‐year payout period might be too short. A recent study of Singaporeans with acquired disability found that one‐third of those with 3 or more ADL limitations were still alive 6 years later.^124^ While one‐third of ElderShield enrollees have supplementary coverage, details of their benefit levels and payout duration have not been published, and the adequacy of their insurance plans cannot be empirically assessed. One way to raise the cash benefits is by extending the pay‐in period. In some countries that rely on social long‐term care insurance, such as Germany and the Netherlands, people are enrolled as soon as they enter the workforce.^125^, ^126^, ^127^ Faced with growing fiscal pressure, policymakers in Japan are considering a policy revision that would require contributions to their social LTCI program to start at age 20 instead of 40.^128^ The Singapore government chose to start automatic enrollment for ElderShield at age 40 in an effort to minimize opt‐outs, as younger adults are thought to be more prone to future discounting. However, clear evidence for or against this assumption is lacking.^129^ Therefore, lowering the automatic enrollment age remains a potential strategy to raise prefunding levels in Singapore.

Although it offers flexibility, ElderShield's cash benefit has drawbacks. In countries like the United States, where service benefits are the norm, LTCI plans are designed to pay up to a set dollar limit per day for services. Accordingly, insurers take on most of the risk of price and quality inflation. In Singapore, ElderShield's cash benefit means that insurers do not have to take on that risk. This lack of inflation protection greatly affects the enrollees, as most of them are expected to claim benefits 20 or more years in the future. We conducted simulations (available upon request) showing that at its current level, the basic ElderShield benefit will offset 6% to 9% of estimated unsubsidized monthly LTSS expense in 2037, down from 11% to 16% today. Even though the government reviews ElderShield policies regularly and has raised basic ElderShield coverage from S$300 (US$221) to S$400 (US$294) per month and extended the payout period from 5 to 6 years,^85^, ^88^, ^130^ inflation remains a problem that has not been fully addressed.

Even with automatic enrollment, not every Singaporean aged 40 and over is covered under ElderShield. We estimate that up to 27% of 40‐year‐olds are uninsured each year because of preexisting disabilities, nonmembership in CPF, and opt‐out at enrollment age. Informal workers who do not have Medisave accounts and those with too little saved up, such as women who drop out of the labor market early, run the risk of lapsing even if they were initially enrolled. Others choose to opt out after being automatically enrolled. If the government wants the LTSS expenses of the most vulnerable people to be partly financed through LTCI, it will have to consider providing them with premium support, as it now does for those covered by the MediShield Life program. Public education is also needed to heighten people's awareness of their potential LTSS financing needs and to help inform their choice of benefit levels. These all are known challenges that the ongoing review of ElderShield will attempt to address. The appointed committee is expected to announce its conclusions and recommendations by the end of 2017.^131^ Singapore's relatively recent LTSS financing system certainly is still a work in progress, but the way it combines tiered, means‐tested public subsidies to LTCI and other private resources is a valuable idea that other countries might consider.

## LTSS Financing Ideas From Singapore

The long‐running discourse about how to finance long‐term care for elderly Americans has continued after the repeal of the CLASS Act.^6^, ^132^, ^133^, ^134^, ^135^ The CLASS insurance program was ultimately not implemented because there was uncertainty regarding the program's long‐term financial sustainability owing to its vulnerability to adverse selection.^136^ The concern was that the resulting upward pressure on premium levels, which were already considered too high, would create an insurance death spiral.^137^, ^138^

Independent of CLASS's shortcomings, the LTCI markets face several well‐documented challenges. Large intertemporal risks lead to high transaction costs, which in turn give rise to expensive insurance plans.^139^, ^140^, ^141^, ^142^ Asymmetric information results in adverse selection.^139^, ^141^, ^142^, ^143^ In addition, consumer irrationality in the forms of myopia, underestimation of one's risk of needing LTSS, narrow framing, and bounded rationality further contribute to the low uptake of LTCI.^1^, ^144^, ^145^, ^146^, ^147^, ^148^ In regard to bounded rationality, many Americans are unaware that LTCI exists, that they are eligible for coverage, or that their existing health care insurance plans do not cover LTSS.^144^, ^149^ Complexity in the myriad of LTCI plans on offer also deter some American consumers from getting coverage, as they are overwhelmed and confused by the marketing information.^146^ While ElderShield shares some design features with CLASS, such as early enrollment and the offer of cash benefits, its implementation is very different.

First, the Singapore government sets clear expectations about the role of ElderShield. In the United States, buyers of LTCI see being insured as a way to protect their assets, financial independence, and living standards.^150^ Consequently, LTCI benefits must be sufficiently large in order to match higher LTSS costs in the future. Indeed, the average annual premium of LTCI plans in the individual market has been rising and was US$2,283 in 2010.^150^ This mind‐set is not mainstream in Singapore, however. The policy goal for ElderShield has never been to be the primary payer for LTSS expenses.^75^ Rather, it is designed to augment individuals’ own resources, both social and financial, as well as public subsidies in defraying LTSS expenses.^75^ The Singapore system does not provide full financial protection against LTSS expenses but instead uses LTCI to reduce the financial burden, whereas public subsidies provide additional help to low‐income individuals. Thus, the government chose a relatively low monthly benefit as the default for basic ElderShield coverage. As a result, basic LTSS coverage can be bought from private insurers at correspondingly affordable premium rates (US$129 to US$160 per year). The provision of basic coverage has the added benefits of raising awareness and highlighting coverage shortfalls to potential buyers of supplemental LTCI.

Second, enrollment in ElderShield is subject to medical underwriting. One of the design features leading to high premium estimates in the CLASS program is the lack of medical underwriting.^23^ In contrast, affordability of ElderShield premiums for basic coverage is enhanced by excluding people with serious preexisting ADL limitations from the risk pool. Individuals with certain chronic medical conditions can enroll in ElderShield if they accept higher premium loads. Such medical underwriting is pragmatic, since insurance is meant to provide support when low‐probability, high‐impact events occur. It is more expensive and less efficient to insure people whose probability of needing LTSS is known to be high. This practice is not at odds with the policy principles articulated by the US Senate Committee on Long‐Term Care. Its report states that “an effective, publicly‐funded safety net is essential for those with limited lifetime resources, including those whose physical, intellectual, or cognitive disability originate early in life.”^3^ By creating a separate long‐term care financing program for people with disabilities, based on government subsidies, charitable donations, and savings (both private and CPF balances), Singapore is able to keep ElderShield premiums affordable.

Third, to contain adverse selection, the enrollment age is set at 40 years. This is comparable to the average enrollment age in France (40 to 44 years^5^) and is lower than that in the United States, where 24.7% of new LTCI enrollees applied between the ages of 45 and 54, and 54% applied between the ages of 55 and 64.^13^ Early enrollment has the additional benefit of increased time for prefunding, as well as prefunding during people's most productive years. Since ElderShield premiums have been stable since its launch and are payable for only 25 years from age 40, enrollees have not had to face the uncertainty of rate hikes or the burden of premium payments in their retirement years. Further, unlike CLASS, which has a 5‐year waiting period, ElderShield enrollees may make claims at any time after enrollment. Together, these attributes help make ElderShield coverage a palatable default option.

Fourth, the automatic enrollment feature of ElderShield circumvents issues related to consumer irrationality. By not requiring much or any action on the enrollees’ part, the government no longer has to rely on individuals to overcome their tendencies to procrastinate and to avoid contemplating unpleasant future events. In the behavioral economics literature, such use of the default option is referred to as a “nudge.”^151^ While automatic enrollment is likely the main driver of the high rate of ElderShield coverage in Singapore, it is not sufficient by itself. By making premium payment automatic and relatively painless for Singaporeans through the use of CPF funds, the government also effectively decreases the salience of this expense, thereby reducing the likelihood of opt‐out among those actively exercising their ability to choose. Note that salience reduction is another application of behavioral economics principles that aim at influencing behaviors.^152^ Even though the United States does not have the equivalent of a CPF system, allowing Americans to tap their 401(k)/403(b) accounts, individual retirement accounts (IRA), or Section 125 accounts to pay their LTCI premiums may enable automatic enrollment and lessen the impact on their levels of disposable income, thereby encouraging more people to buy LTCI. The downside of such an approach would be a reduction in retirement savings. This concern may be mitigated by requiring the maintenance of minimum retirement account balances and setting withdrawal limits. An alternative path to automatic enrollment in the United States may be through the development of employer‐sponsored LTCI programs by modeling incentives and regulations on those that are currently in place for retirement savings programs. These ideas are consistent with recommendations from both the BPC and the SCAN Foundation.^2^, ^24^ The Singapore case demonstrates such recommendations are actionable and may have desirable outcomes in terms of LTCI uptake.

Fifth, ElderShield payout in the form of defined cash benefits addresses the issue of consumer bounded rationality because it reduces the complexity of LTCI plans for consumers, a problem that has plagued LTCI markets in the United States for years. In the United States, however, insurers tend to offer service benefits, as claim rates are perceived to be higher with cash benefits.^95^ Another issue is that the cash benefit may not be spent on LTSS but on other goods instead. However, the practice of offering LTCI benefits in cash is not new; a number of European countries have managed cash benefit systems for years.^153^ This model gained traction in the United States after the first Cash and Counseling demonstration projects were conducted in 1998.^154^ Today, such participant‐directed programs that disburse Medicaid long‐term care benefits as cash to older people are available in all 50 states and the District of Columbia.^155^ In Singapore, ElderShield payouts may be disbursed to the beneficiaries, to their caregivers (if the beneficiaries are medically certified to lack mental capacity), or to LTSS providers directly. Strict eligibility criteria and the requirement of evaluation by accredited independent assessors minimize the possibility of fraud. When payouts are disbursed to caregivers, they are committed, in writing, to use the cash only for the care and benefit of the beneficiaries.^156^ Since public financial assistance for LTSS is limited and is provided only after applicants demonstrate that they have exhausted all other possible sources of funding, ElderShield benefits are less likely to be misused.

Last, a possible reason cited for the small LTCI market in the United States is crowd‐out by substitutes such as readily available informal care,^157^ and alternative LTSS funding sources such as home equity^158^ or Medicaid.^143^, ^159^ Some level of crowd‐out could exist in Singapore, although the phenomenon has not been well studied. However, instead of crowding out LTCI, Singapore's tiered public subsidy structure may be complementary to the development and maintenance of an LTCI market. Those with a low MPCHI see the benefit of retaining inexpensive basic ElderShield coverage because the cash payout does defray a meaningful portion of their postsubsidy OOP expense. People with a moderate MPCHI are better able to afford supplemental plans and may be motivated to buy more coverage, since they face proportionally higher postsubsidy OOP expenses. Finally, LTCI is one source of financial protection for those who are ineligible for public subsidies. Accordingly, LTCI can appeal to individuals along the entire income distribution.

Debates about optimal long‐term care financing policy through insurance contain an inherent tension between the breadth and depth of coverage. The former is indicated by LTCI take‐up rates; the latter is measured by the degree of financial protection LTCI provides. If one wants to limit budgetary pressure posed by public LTSS financing, the problem becomes one of maximizing private contributions to LTCI by those who can afford it so that public funds can be directed at those who cannot. By this formulation, the total private contribution to be maximized is the product of the take‐up rate and the depth of coverage, as the latter is proportional to LTCI premiums. The Singapore LTCI system can be described as one in which high take‐up is achieved at the expense of depth of coverage. A moderate increase in basic coverage is unlikely to negatively affect take‐up rates and is likely one of the goals of the policy reform currently under way. The United States likely lies, however, on the other side of this trade‐off, as lowering insurance coverage would likely increase take‐up rates and total private contributions to LTCI.

US policymakers face the challenge of integrating elements of private LTCI and social insurance programs to meet the nation's long‐term care financing needs. The Singapore case illustrates how LTSS can be financed through a mix of public and private sources comprising tiered, means‐tested public subsidies and government grants to LTSS providers that lower LTSS prices, charitable donations, voluntary LTCI, and other private resources from the care recipient and his or her family. While the optimal mix of private and public options will ultimately depend on the national and political context of the United States, it is worth noting that hybrid financing systems like Singapore's can be implemented incrementally. This is especially relevant to the United States, given its current polarized political context that makes finding a compromise on any far‐reaching reform extremely unlikely.
